# Chlorido[1-(2-oxidophen­yl)ethyl­idene][tris­(3,5-dimethyl­pyrazol-1-yl)hydro­borato]iridium(III) chloro­form monosolvate

**DOI:** 10.1107/S1600536813007344

**Published:** 2013-03-23

**Authors:** Laura L. Santos, Margarita Paneque, Kurt Mereiter

**Affiliations:** a Instituto de Investigaciones Químicas (IIQ) and Departamento de Química Inorgánica, Consejo Superior de Investigaciones Cientificas (CSIC) and Universidad de Sevilla, Avenida Américo Vespucio 49, 41092 Sevilla, Spain; b Institute of Chemical Technologies and Analytics, Vienna University of Technology, Getreidemarkt 9/164SC, A-1060 Vienna, Austria

## Abstract

In the title compound, [Ir(C_15_H_22_BN_6_)(C_8_H_7_O)Cl]·CHCl_3_, the Ir atom is formally trivalent and is coordinated in a slightly distorted octa­hedral geometry by three facial N atoms, one C atom, one O atom and one Cl atom. The Ir=C_carbene_ bond is strong and short and exerts a notable effect on the *trans*-Ir—N bond, which is about 0.10 Å longer than the two other Ir—N bonds. The chloro­form solvent mol­ecule is anchored *via* a weak C—H⋯Cl hydrogen bond to the Cl atom of the Ir complex mol­ecule. In the crystal, the constituents adopt a layer-like arrangement parallel to (010) and are held together by weak inter­molecular C—H⋯Cl hydrogen bonds, as well as weak Cl⋯Cl [3.498 (2) Å] and Cl⋯π [3.360 (4) Å] inter­actions. A weak intra­molecular C—H⋯O hydrogen bond is also observed.

## Related literature

The title compound represents a well crystallizing air-stable chloro­form solvate of a mononuclear iridium complex based on the (hydrogen tris­(3,5-dimethyl­pyrazol­yl)borate-*N*,*N*′,*N*′′)-iridium moiety Ir[Tp^Me2^]. Its formation from [(Tp^Me2^)Ir(C_6_H_5_)_2_(*k* ^1^-N_2_)] (C_6_H_5_ = phenyl, N_2_ = dinitro­gen) and eth­oxy­benzene involved multiple C—C,H,O,Cl bond transformations by the outstanding activity of the Ir[Tp^Me2^] moiety. For general information on C—H and C—C activation, see: Lin & Yamamoto (1999 ▶); Dyker (1999 ▶); Labinger & Bercaw (2002 ▶). For C—H bond activation reactions of ethers by Ir[Tp^Me2^] complexes, see: Lara *et al.* (2009 ▶); Conejero *et al.* (2010 ▶); Santos *et al.* (2013 ▶). For the synthesis of the complex and related crystal structures, see: Gutiérrez-Puebla *et al.* (1998 ▶); Lara *et al.* (2009 ▶). For a description of the Cambridge Structural Database, see: Allen (2002 ▶). For bond-length data, see: Allen *et al.* (1987 ▶).
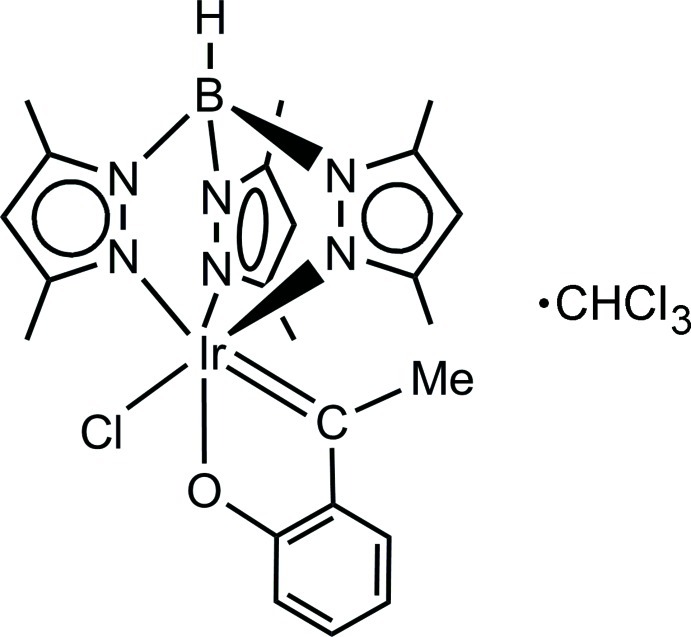


## Experimental

### 

#### Crystal data

[Ir(C_15_H_22_BN_6_)(C_8_H_7_O)Cl]·CHCl_3_
*M* *_r_* = 763.35
Monoclinic, 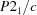

*a* = 10.1271 (4) Å
*b* = 19.1711 (8) Å
*c* = 14.3154 (6) Å
β = 91.956 (2)°
*V* = 2777.7 (2) Å^3^
*Z* = 4
Mo *K*α radiation
μ = 5.22 mm^−1^
*T* = 173 K
0.32 × 0.15 × 0.10 mm

#### Data collection

Bruker SMART APEX CCD diffractometer
Absorption correction: multi-scan (*SADABS*; Bruker, 2003 ▶) *T*_min_ = 0.343, *T*_max_ = 0.593
52411 measured reflections
8053 independent reflections
6999 reflections with *I* > 2σ(*I*)
*R*_int_ = 0.037

#### Refinement

*R*[*F* ^2^ > 2σ(*F* ^2^)] = 0.028
*wR*(*F* ^2^) = 0.071
*S* = 1.02
8053 reflections
341 parameters
H-atom parameters constrained
Δρ_max_ = 1.29 e Å^−3^
Δρ_min_ = −1.43 e Å^−3^

### 

Data collection: *SMART* (Bruker, 2003 ▶); cell refinement: *SAINT* (Bruker, 2003 ▶); data reduction: *SAINT*; program(s) used to solve structure: *SHELXS97* (Sheldrick, 2008 ▶); program(s) used to refine structure: *SHELXL97* (Sheldrick, 2008 ▶); molecular graphics: *Mercury* (Macrae *et al.*, 2006 ▶); software used to prepare material for publication: *PLATON* (Spek, 2009 ▶) and *publCIF* (Westrip, 2010 ▶).

## Supplementary Material

Crystal structure: contains datablock(s) I, global. DOI: 10.1107/S1600536813007344/lh5594sup1.cif

Structure factors: contains datablock(s) I. DOI: 10.1107/S1600536813007344/lh5594Isup2.hkl

Additional supplementary materials:  crystallographic information; 3D view; checkCIF report

## Figures and Tables

**Table 1:** Selected bond lengths (Å)

Ir1—C22	1.937 (3)
Ir1—N3	2.056 (3)
Ir1—N1	2.059 (3)
Ir1—O1	2.063 (2)
Ir1—N5	2.155 (3)
Ir1—Cl1	2.3500 (8)

**Table 2:** Hydrogen-bond geometry (Å, °)

*D*—H⋯*A*	*D*—H	H⋯*A*	*D*⋯*A*	*D*—H⋯*A*
C24—H24⋯Cl1	1.00	2.55	3.488 (5)	156
C11—H11*A*⋯O1	0.98	2.37	3.230 (4)	146
C11—H11*C*⋯Cl3^i^	0.98	2.65	3.609 (4)	166

Symmetry code: (i) 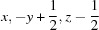.

**Fig. 1.**
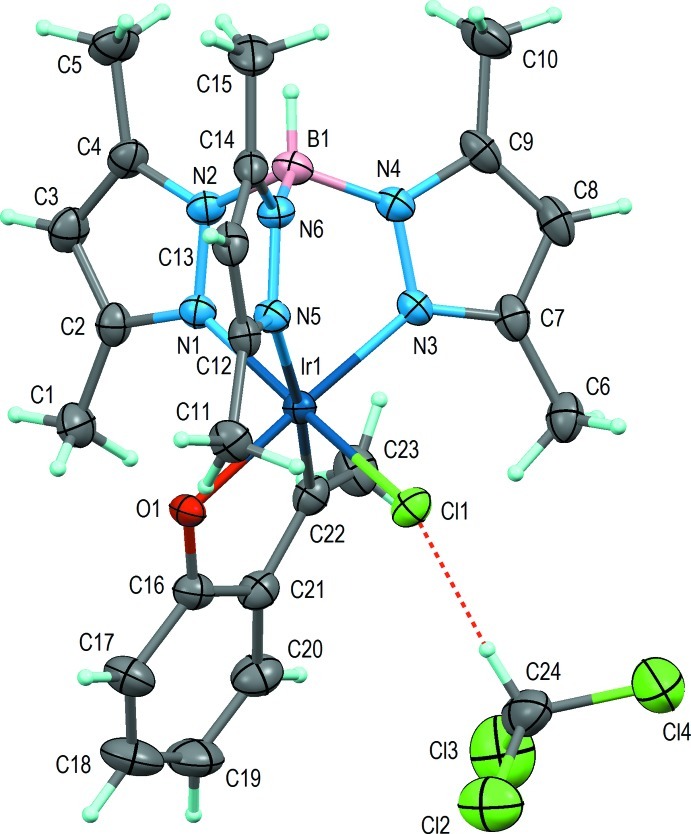
The asymmetric unit of the title structure 1.CHCl3 with displacement ellipsoids drawn at the 50% probability level and the hydrogen bond C24—H24···Cl1 as a dashed red line.

**Fig. 2.**
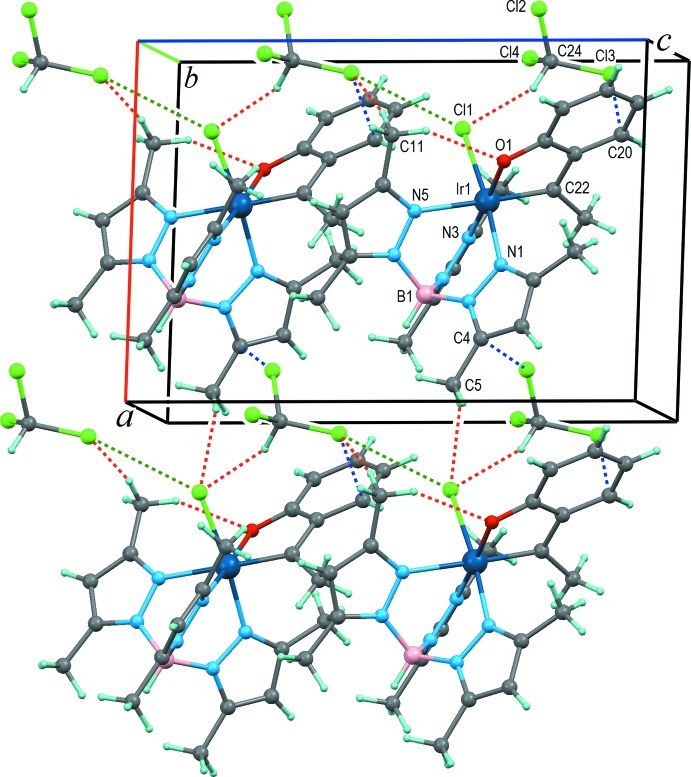
View of the structure of 1.CHCl3 approximately along [010] in the range 0 < y < 1/2; showing the interactions C—H···O,Cl (red), Cl···Cl (green), and Cl···π (blue) as dashed lines.

**Fig. 3.**
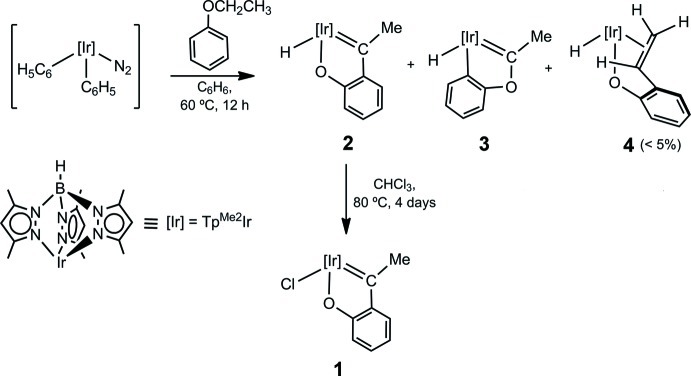
Reaction scheme for the synthesis of 1.

## supplementary crystallographic information

### Comment

Transition metal compounds capable of inducing C—H bond activation and subsequent C—C bond formation have important applications in the synthesis of complex organic molecules from simple, commonly available substrates (Lin & Yamamoto, 1999; Dyker, 1999; Labinger & Bercaw, 2002). Iridium complexes with hydrogen-tris(pyrazolyl)borate as a stabilizing ligand and labile coordination sites have been found to show an outstanding potential at this respect (Conejero *et al.*, 2010). Part of our work in this field has derived from the study of reactions of ethers with reactive Ir complexes coordinated by the hydrogen-tris(3,5-dimethylpyrazolyl)borate ligand (Tp^Me2^) (Lara *et al.*, 2009; Conejero *et al.*, 2010; Santos *et al.*, 2013). When the complex [(Tp^Me2^)Ir(C_6_H_5_)_2_(*k*^1^-N_2_)] (C_6_H_5_ = phenyl, N_2_ = dinitrogen; Gutiérrez-Puebla *et al.*, 1998) and C_6_H_5_OCH_2_CH_3_ (ethoxybenzene) are heated to 333K in cyclohexane a mixture of three compounds is formed (see reaction scheme Fig. 3). The major reaction product is 2, the precursor of the title complex 1. Compound 2 is a hydride-alkylidene whose formation requires multiple C—H bond activations, C—O bond cleavage and C—C bond formation. The other reaction products are a heteroatom-stabilized hydride-carbene 3 derived from three C—H activations of the organic product, and the minor reaction product 4 (*ca* 5%), which is the hydride-alkene tautomer of 2. Compound 2 could be prepared independently in nearly quantitative yield (~95%) by the reaction of [(Tp^Me2^)Ir(C_6_H_5_)_2_(*k*^1^-N_2_)] with 2-ethylphenol (Lara *et al.*, 2009). Compound 2 is stable at room temperature but at higher temperatures is in equilibrium with compound 4. On the other hand, if 2 is heated in chloroform at 353K for 4 days, a C—Cl bond activation takes place whereby the hydride is exchanged against a Cl atom under concomitant formation of dichloromethane. The resulting complex 1 (Fig. 3) crystallizes from the excess of CHCl_3_ under formation of the title compound, an air-stable solvate 1.CHCl_3_, (I). In 1 the iridium atom exhibits a relatively regular octahedral coordination by three pyrazole nitrogen atoms, the carbene atom C22, the phenolate oxygen O1, and the chloride ligand Cl1 (Fig. 1). The *cis* bond angles about Ir vary from 82.18 (12)° (C22—Ir1—O1) to 98.38 (13)° (C22—Ir1—N3) and the *trans* bond angles from 173.11 (8)° to 177.55 (9)° (N3—Ir1—O1). The metal-carbene bond Ir1—C22 = 1.937 (3) Å is characteristically short and in good accord with Ir-carbene bonds of well refined crystal structures in the Cambridge Structural Database (version 5.33; Allen, 2002), which gave a mean value of 1.942 (64) Å for 57 crystal structures with 69 bonds. The Ir—N bonds (Table 1) show a typical elongation of *ca* 0.1 Å for the bond Ir1—N5 *trans* to the carbene ligand. The Ir—O and Ir—Cl bonds adopt normal values (Allen *et al.*, 1987). Bond lengths and angles in the Tp^Me2^ ligand compare well with related complexes (*e.g.*: Lara *et al.*, 2009; Santos *et al.*, 2013). The chelate ring formed by the carbene ligand has a flat envelope conformation with O1—C16—C21—C22 perfectly planar (r.m.s. deviation from planarity 0.0002 Å) and Ir1 displaced from this plane by -0.352 (5) Å whereas the terminal methyl carbon C23 is 0.419 (7) Å off from this plane. The carbene atom C22 has a very flat pyramidal coordination and deviates by -0.064 (4) Å from the plane defined by Ir1, C21, and C23. In the crystal structure the Ir complexes 1 and the CHCl_3_ molecules are organized in a layer-like fashion parallel to (010) as shown in Fig. 2. Such layers are centered at *y*≈ 1/4; and *y*≈ 3/4;. The CHCl_3_ molecule is anchored in the structure *via* a pronounced C—H···Cl hydrogen bond (C···Cl = 3.360 (4) Å) to the Cl1 atom of the Ir complex (Fig. 1 and Table 2). It is moreover fixed by the interaction C11—H11*c*···Cl3 [C11(*x*,1/2 - *y*,1/2 + *z*)···Cl3 = 3.608 (4) Å], by the halogen-halogen contact Cl3···Cl1(*x*,1/2 - *y*,1/2 + *z*) = 3.498 (2) Å, and two side-on contacts between the π-orbitals of arene rings and Cl [Cl2 with pyrazole ring 1 and the shortest contact distance Cl2···C4(*x* - 1,*y*,*z*) = 3.360 (4) Å; Cl3 with the phenyl ring and the shortest contact distance Cl3···C20 = 3.396 (4) Å]. These interactions are included in Fig. 2. Interactions between the Ir complexes are unremarkable and consist essentially of van der Waals contacts. The interaction C11—H11a···O3 is intramolecular.

### Experimental

A solution of 2 (0.030 g, 0.049 mmol; see Fig. 3; for synthesis see Lara *et al.*, 2009) in CHCl_3_ (3 ml) was stirred at 353K for 4 days. After this time the solvent was removed under reduced pressure. NMR spectra of the crude product revealed the presence of complex 1 in 70% spectroscopic yield. Crystallization from pentane/CH_2_Cl_2_/CHCl_3_ at 253K gave compound 1 as a dark green microcrystalline solid. ^1^H NMR (CDCl_3_, 298 K) δ 7.47, 7.28, 7.19, 6.60 (dd, ddd, d, ddd, 1 H each, ^3^*J*_HH_≈ 8.5, ^4^*J*_HH_≈1 Hz, 4 CH_ar_), 5.89, 5.88, 5.50 (s, 1 H each, 3 CH_pz_), 3.07 (s, 3 H, Ir═CCH_3_), 2.77, 2.49, 2.40, 2.36, 2.32, 1.32 (s, 3 H each, 6 Me_pz_). ^13^C{^1^H} NMR (CDCl_3_, 25 °C) δ 273.2(Ir═C), 192.5 (Ir—O—C), 154.7, 153.8, 153.3, 152.3, 144.6, 144.2, 144.1 (Ir ═ C-*C* + C_qpz_), 141.0, 124.2, 119.7, 115.7 (CH_ar_), 108.4, 108.3, 108.2 (CH_pz_), 34.0 (Ir=C*C*H_3_), 16.4, 14.1, 13.1, 13.0, 12.4, 12.2 (Me_pz_). Crystals of 1.CHCl_3_ for X-ray diffraction were obtained by recrystallization from CHCl_3_/pentane.

### Refinement

H atoms were placed in calculated positions and thereafter treated as riding, C—H = 0.95–1.00 Å, B—H = 1.00 Å, *U*i_so_(H) = 1.2–1.5*U*_eq_(C,B), using AFIX 137 of program *SHELXL97* (Sheldrick, 2008) for the methyl groups.

### Crystal data

**Table:**

[Ir(C_15_H_22_BN_6_)(C_8_H_7_O)Cl]·CHCl_3_	*F*(000) = 1496
*M**_r_* = 763.35	*D*_x_ = 1.825 Mg m^−^^3^
Monoclinic, *P*2_1_/*c*	Mo *K*α radiation, λ = 0.71073 Å
Hall symbol: -P 2ybc	Cell parameters from 8986 reflections
*a* = 10.1271 (4) Å	θ = 2.3–30.0°
*b* = 19.1711 (8) Å	µ = 5.22 mm^−^^1^
*c* = 14.3154 (6) Å	*T* = 173 K
β = 91.956 (2)°	Irregular, dark green
*V* = 2777.7 (2) Å^3^	0.32 × 0.15 × 0.10 mm
*Z* = 4	

### Data collection

**Table:**

Bruker SMART APEX CCD diffractometer	8053 independent reflections
Radiation source: fine-focus sealed tube	6999 reflections with *I* > 2σ(*I*)
Graphite monochromator	*R*_int_ = 0.037
ω and φ scans	θ_max_ = 30.0°, θ_min_ = 2.6°
Absorption correction: multi-scan (*SADABS*; Bruker, 2003)	*h* = −14→14
*T*_min_ = 0.343, *T*_max_ = 0.593	*k* = −26→26
52411 measured reflections	*l* = −20→20

### Refinement

**Table:**

Refinement on *F*^2^	Primary atom site location: structure-invariant direct methods
Least-squares matrix: full	Secondary atom site location: difference Fourier map
*R*[*F*^2^ > 2σ(*F*^2^)] = 0.028	Hydrogen site location: inferred from neighbouring sites
*wR*(*F*^2^) = 0.071	H-atom parameters constrained
*S* = 1.02	*w* = 1/[σ^2^(*F*_o_^2^) + (0.038*P*)^2^ + 4.5744*P*] where *P* = (*F*_o_^2^ + 2*F*_c_^2^)/3
8053 reflections	(Δ/σ)_max_ = 0.001
341 parameters	Δρ_max_ = 1.29 e Å^−^^3^
0 restraints	Δρ_min_ = −1.43 e Å^−^^3^

### Special details

**Table:**

Geometry. All e.s.d.'s (except the e.s.d. in the dihedral angle between two l.s. planes) are estimated using the full covariance matrix. The cell e.s.d.'s are taken into account individually in the estimation of e.s.d.'s in distances, angles and torsion angles; correlations between e.s.d.'s in cell parameters are only used when they are defined by crystal symmetry. An approximate (isotropic) treatment of cell e.s.d.'s is used for estimating e.s.d.'s involving l.s. planes.
Refinement. Refinement of *F*^2^ against ALL reflections. The weighted *R*-factor *wR* and goodness of fit *S* are based on *F*^2^, conventional *R*-factors *R* are based on *F*, with *F* set to zero for negative *F*^2^. The threshold expression of *F*^2^ > σ(*F*^2^) is used only for calculating *R*-factors(gt) *etc*. and is not relevant to the choice of reflections for refinement. *R*-factors based on *F*^2^ are statistically about twice as large as those based on *F*, and *R*- factors based on ALL data will be even larger.

### Fractional atomic coordinates and isotropic or equivalent isotropic displacement parameters (Å^2^)

**Table:**

	*x*	*y*	*z*	*U*_iso_*/*U*_eq_	
Ir1	0.436291 (10)	0.157920 (6)	0.682819 (7)	0.01911 (4)	
Cl1	0.23334 (8)	0.20336 (4)	0.62640 (6)	0.02847 (15)	
B1	0.6944 (4)	0.16488 (19)	0.5669 (3)	0.0262 (7)	
H0B	0.7782	0.1680	0.5322	0.031*	
N1	0.6159 (3)	0.11309 (14)	0.71808 (18)	0.0227 (5)	
N2	0.7168 (3)	0.12094 (14)	0.65687 (19)	0.0247 (5)	
N3	0.5334 (3)	0.24641 (14)	0.64204 (18)	0.0234 (5)	
N4	0.6481 (3)	0.23785 (15)	0.59388 (19)	0.0257 (5)	
N5	0.4663 (3)	0.11764 (14)	0.54460 (18)	0.0225 (5)	
N6	0.5867 (3)	0.12870 (14)	0.50605 (18)	0.0236 (5)	
O1	0.3316 (2)	0.07061 (11)	0.72065 (14)	0.0216 (4)	
C1	0.5825 (4)	0.0473 (2)	0.8677 (3)	0.0357 (8)	
H1A	0.5529	0.0884	0.9018	0.054*	
H1B	0.6378	0.0181	0.9095	0.054*	
H1C	0.5055	0.0205	0.8451	0.054*	
C2	0.6607 (3)	0.06994 (17)	0.7866 (2)	0.0267 (6)	
C3	0.7896 (3)	0.05026 (19)	0.7680 (3)	0.0323 (7)	
H3	0.8448	0.0201	0.8048	0.039*	
C4	0.8217 (3)	0.08277 (18)	0.6863 (2)	0.0281 (7)	
C5	0.9489 (3)	0.0795 (2)	0.6354 (3)	0.0380 (8)	
H5A	0.9295	0.0740	0.5683	0.057*	
H5B	1.0014	0.0397	0.6583	0.057*	
H5C	0.9989	0.1227	0.6464	0.057*	
C6	0.3925 (4)	0.34615 (18)	0.6934 (3)	0.0340 (7)	
H6A	0.3425	0.3095	0.7241	0.051*	
H6B	0.3359	0.3689	0.6456	0.051*	
H6C	0.4224	0.3807	0.7399	0.051*	
C7	0.5096 (4)	0.31472 (17)	0.6486 (2)	0.0277 (6)	
C8	0.6101 (4)	0.35093 (18)	0.6050 (3)	0.0337 (8)	
H8	0.6178	0.4001	0.5992	0.040*	
C9	0.6957 (4)	0.30165 (18)	0.5719 (2)	0.0308 (7)	
C10	0.8185 (4)	0.3117 (2)	0.5176 (3)	0.0454 (10)	
H10A	0.8053	0.2917	0.4550	0.068*	
H10B	0.8929	0.2884	0.5501	0.068*	
H10C	0.8374	0.3617	0.5124	0.068*	
C11	0.2581 (3)	0.05407 (19)	0.5004 (3)	0.0308 (7)	
H11A	0.2475	0.0463	0.5674	0.046*	
H11B	0.2415	0.0104	0.4664	0.046*	
H11C	0.1952	0.0897	0.4781	0.046*	
C12	0.3956 (3)	0.07824 (16)	0.4843 (2)	0.0232 (6)	
C13	0.4714 (4)	0.06377 (17)	0.4063 (2)	0.0280 (6)	
H13	0.4455	0.0369	0.3530	0.034*	
C14	0.5906 (3)	0.09640 (17)	0.4225 (2)	0.0263 (6)	
C15	0.7068 (4)	0.1003 (2)	0.3615 (3)	0.0387 (9)	
H15A	0.7034	0.0615	0.3169	0.058*	
H15B	0.7884	0.0973	0.4001	0.058*	
H15C	0.7049	0.1446	0.3274	0.058*	
C16	0.2741 (3)	0.08151 (19)	0.7994 (2)	0.0280 (6)	
C17	0.1875 (4)	0.0306 (2)	0.8350 (3)	0.0377 (8)	
H17	0.1654	−0.0100	0.7999	0.045*	
C18	0.1364 (4)	0.0413 (3)	0.9210 (3)	0.0500 (11)	
H18	0.0767	0.0077	0.9443	0.060*	
C19	0.1686 (4)	0.1001 (3)	0.9767 (3)	0.0493 (11)	
H19	0.1324	0.1054	1.0366	0.059*	
C20	0.2535 (4)	0.1500 (2)	0.9430 (3)	0.0384 (8)	
H20	0.2762	0.1896	0.9799	0.046*	
C21	0.3070 (3)	0.14175 (19)	0.8528 (2)	0.0282 (7)	
C22	0.4044 (3)	0.18475 (18)	0.8105 (2)	0.0263 (6)	
C23	0.4790 (4)	0.2367 (2)	0.8696 (3)	0.0356 (8)	
H23A	0.4192	0.2741	0.8877	0.053*	
H23B	0.5510	0.2563	0.8340	0.053*	
H23C	0.5156	0.2136	0.9258	0.053*	
C24	0.0420 (5)	0.2910 (2)	0.7869 (4)	0.0508 (11)	
H24	0.1171	0.2666	0.7573	0.061*	
Cl2	−0.10099 (13)	0.24331 (7)	0.76482 (9)	0.0592 (3)	
Cl3	0.07774 (17)	0.29868 (9)	0.90450 (11)	0.0756 (4)	
Cl4	0.02838 (17)	0.37582 (8)	0.73962 (13)	0.0804 (5)	

### Atomic displacement parameters (Å^2^)

**Table:**

	*U*^11^	*U*^22^	*U*^33^	*U*^12^	*U*^13^	*U*^23^
Ir1	0.01915 (6)	0.01930 (6)	0.01887 (6)	−0.00133 (4)	0.00052 (4)	−0.00196 (4)
Cl1	0.0237 (3)	0.0308 (4)	0.0307 (4)	0.0028 (3)	−0.0012 (3)	−0.0030 (3)
B1	0.0226 (16)	0.0281 (18)	0.0280 (17)	−0.0040 (13)	0.0031 (13)	0.0005 (14)
N1	0.0205 (12)	0.0230 (12)	0.0247 (12)	−0.0017 (10)	0.0005 (9)	0.0001 (10)
N2	0.0187 (12)	0.0266 (13)	0.0285 (13)	−0.0012 (10)	−0.0002 (10)	0.0004 (10)
N3	0.0240 (12)	0.0219 (12)	0.0245 (12)	−0.0032 (10)	0.0014 (10)	0.0012 (10)
N4	0.0244 (13)	0.0256 (13)	0.0273 (13)	−0.0041 (10)	0.0022 (10)	0.0008 (10)
N5	0.0227 (12)	0.0223 (12)	0.0225 (12)	−0.0025 (10)	0.0020 (9)	−0.0005 (10)
N6	0.0229 (12)	0.0247 (13)	0.0234 (12)	0.0006 (10)	0.0048 (10)	−0.0001 (10)
O1	0.0229 (10)	0.0216 (10)	0.0207 (10)	−0.0041 (8)	0.0053 (8)	−0.0022 (8)
C1	0.0391 (19)	0.0357 (19)	0.0325 (18)	0.0050 (15)	0.0022 (14)	0.0104 (15)
C2	0.0289 (16)	0.0243 (15)	0.0264 (15)	0.0011 (12)	−0.0046 (12)	0.0003 (12)
C3	0.0267 (16)	0.0341 (18)	0.0355 (18)	0.0043 (13)	−0.0072 (13)	0.0021 (14)
C4	0.0199 (14)	0.0273 (16)	0.0370 (17)	−0.0013 (12)	−0.0032 (12)	−0.0029 (13)
C5	0.0191 (15)	0.046 (2)	0.049 (2)	0.0000 (14)	−0.0007 (14)	−0.0016 (17)
C6	0.046 (2)	0.0254 (17)	0.0305 (17)	0.0046 (14)	−0.0010 (15)	−0.0034 (13)
C7	0.0384 (18)	0.0210 (14)	0.0234 (15)	−0.0032 (13)	−0.0034 (12)	−0.0021 (12)
C8	0.048 (2)	0.0218 (16)	0.0310 (17)	−0.0099 (14)	−0.0032 (15)	0.0013 (13)
C9	0.0359 (18)	0.0272 (16)	0.0291 (16)	−0.0121 (14)	−0.0015 (13)	0.0022 (13)
C10	0.044 (2)	0.042 (2)	0.051 (2)	−0.0161 (18)	0.0114 (19)	0.0053 (19)
C11	0.0297 (16)	0.0307 (17)	0.0316 (17)	−0.0065 (13)	−0.0040 (13)	−0.0030 (13)
C12	0.0288 (15)	0.0188 (13)	0.0217 (14)	−0.0005 (11)	−0.0032 (11)	0.0002 (11)
C13	0.0404 (18)	0.0228 (15)	0.0208 (14)	0.0020 (13)	0.0007 (12)	−0.0010 (11)
C14	0.0353 (17)	0.0212 (14)	0.0226 (14)	0.0042 (12)	0.0051 (12)	0.0015 (11)
C15	0.050 (2)	0.037 (2)	0.0302 (17)	0.0049 (17)	0.0179 (16)	0.0016 (15)
C16	0.0252 (15)	0.0328 (17)	0.0261 (15)	−0.0011 (13)	0.0027 (12)	0.0016 (13)
C17	0.0366 (19)	0.042 (2)	0.0348 (19)	−0.0111 (16)	0.0075 (15)	0.0016 (16)
C18	0.042 (2)	0.069 (3)	0.041 (2)	−0.014 (2)	0.0164 (18)	0.005 (2)
C19	0.042 (2)	0.065 (3)	0.042 (2)	−0.001 (2)	0.0193 (18)	0.004 (2)
C20	0.039 (2)	0.049 (2)	0.0279 (17)	0.0034 (17)	0.0046 (14)	−0.0053 (16)
C21	0.0296 (16)	0.0322 (17)	0.0227 (15)	0.0043 (13)	0.0003 (12)	−0.0021 (12)
C22	0.0260 (15)	0.0267 (15)	0.0261 (15)	0.0035 (12)	−0.0019 (12)	−0.0018 (12)
C23	0.047 (2)	0.0311 (18)	0.0280 (17)	−0.0063 (15)	−0.0033 (15)	−0.0064 (14)
C24	0.044 (2)	0.038 (2)	0.070 (3)	0.0005 (18)	0.014 (2)	−0.014 (2)
Cl2	0.0559 (7)	0.0624 (7)	0.0594 (7)	−0.0145 (6)	0.0056 (5)	−0.0200 (6)
Cl3	0.0868 (10)	0.0707 (9)	0.0678 (9)	−0.0084 (8)	−0.0188 (8)	−0.0070 (7)
Cl4	0.0887 (11)	0.0508 (8)	0.1044 (12)	0.0061 (7)	0.0424 (9)	0.0156 (7)

### Geometric parameters (Å, º)

**Table:**

Ir1—C22	1.937 (3)	C8—C9	1.377 (6)
Ir1—N3	2.056 (3)	C8—H8	0.9500
Ir1—N1	2.059 (3)	C9—C10	1.500 (5)
Ir1—O1	2.063 (2)	C10—H10A	0.9800
Ir1—N5	2.155 (3)	C10—H10B	0.9800
Ir1—Cl1	2.3500 (8)	C10—H10C	0.9800
B1—N4	1.529 (5)	C11—C12	1.492 (5)
B1—N6	1.538 (5)	C11—H11A	0.9800
B1—N2	1.549 (5)	C11—H11B	0.9800
B1—H0B	1.0000	C11—H11C	0.9800
N1—C2	1.350 (4)	C12—C13	1.405 (4)
N1—N2	1.376 (4)	C13—C14	1.373 (5)
N2—C4	1.346 (4)	C13—H13	0.9500
N3—C7	1.336 (4)	C14—C15	1.490 (5)
N3—N4	1.380 (4)	C15—H15A	0.9800
N4—C9	1.356 (4)	C15—H15B	0.9800
N5—C12	1.337 (4)	C15—H15C	0.9800
N5—N6	1.372 (3)	C16—C21	1.418 (5)
N6—C14	1.348 (4)	C16—C17	1.419 (5)
O1—C16	1.303 (4)	C17—C18	1.366 (5)
C1—C2	1.492 (5)	C17—H17	0.9500
C1—H1A	0.9800	C18—C19	1.414 (7)
C1—H1B	0.9800	C18—H18	0.9500
C1—H1C	0.9800	C19—C20	1.383 (6)
C2—C3	1.393 (5)	C19—H19	0.9500
C3—C4	1.374 (5)	C20—C21	1.425 (5)
C3—H3	0.9500	C20—H20	0.9500
C4—C5	1.503 (5)	C21—C22	1.435 (5)
C5—H5A	0.9800	C22—C23	1.494 (5)
C5—H5B	0.9800	C23—H23A	0.9800
C5—H5C	0.9800	C23—H23B	0.9800
C6—C7	1.494 (5)	C23—H23C	0.9800
C6—H6A	0.9800	C24—Cl3	1.716 (6)
C6—H6B	0.9800	C24—Cl2	1.732 (5)
C6—H6C	0.9800	C24—Cl4	1.765 (5)
C7—C8	1.397 (5)	C24—H24	1.0000
			
C22—Ir1—N3	98.38 (13)	N3—C7—C6	125.0 (3)
C22—Ir1—N1	93.09 (12)	C8—C7—C6	126.3 (3)
N3—Ir1—N1	89.24 (10)	C9—C8—C7	106.8 (3)
C22—Ir1—O1	82.18 (12)	C9—C8—H8	126.6
N3—Ir1—O1	177.55 (9)	C7—C8—H8	126.6
N1—Ir1—O1	93.12 (9)	N4—C9—C8	107.8 (3)
C22—Ir1—N5	174.27 (12)	N4—C9—C10	122.9 (3)
N3—Ir1—N5	87.20 (10)	C8—C9—C10	129.2 (3)
N1—Ir1—N5	85.68 (10)	C9—C10—H10A	109.5
O1—Ir1—N5	92.29 (9)	C9—C10—H10B	109.5
C22—Ir1—Cl1	93.09 (10)	H10A—C10—H10B	109.5
N3—Ir1—Cl1	91.03 (8)	C9—C10—H10C	109.5
N1—Ir1—Cl1	173.71 (8)	H10A—C10—H10C	109.5
O1—Ir1—Cl1	86.56 (6)	H10B—C10—H10C	109.5
N5—Ir1—Cl1	88.06 (7)	C12—C11—H11A	109.5
N4—B1—N6	109.7 (3)	C12—C11—H11B	109.5
N4—B1—N2	108.9 (3)	H11A—C11—H11B	109.5
N6—B1—N2	107.8 (3)	C12—C11—H11C	109.5
N4—B1—H0B	110.1	H11A—C11—H11C	109.5
N6—B1—H0B	110.1	H11B—C11—H11C	109.5
N2—B1—H0B	110.1	N5—C12—C13	109.3 (3)
C2—N1—N2	107.0 (3)	N5—C12—C11	123.9 (3)
C2—N1—Ir1	134.9 (2)	C13—C12—C11	126.9 (3)
N2—N1—Ir1	117.83 (19)	C14—C13—C12	106.1 (3)
C4—N2—N1	109.6 (3)	C14—C13—H13	127.0
C4—N2—B1	130.4 (3)	C12—C13—H13	127.0
N1—N2—B1	119.9 (3)	N6—C14—C13	107.9 (3)
C7—N3—N4	108.0 (3)	N6—C14—C15	123.0 (3)
C7—N3—Ir1	134.4 (2)	C13—C14—C15	129.1 (3)
N4—N3—Ir1	117.6 (2)	C14—C15—H15A	109.5
C9—N4—N3	108.7 (3)	C14—C15—H15B	109.5
C9—N4—B1	130.7 (3)	H15A—C15—H15B	109.5
N3—N4—B1	120.4 (3)	C14—C15—H15C	109.5
C12—N5—N6	106.9 (2)	H15A—C15—H15C	109.5
C12—N5—Ir1	135.0 (2)	H15B—C15—H15C	109.5
N6—N5—Ir1	117.95 (19)	O1—C16—C21	119.6 (3)
C14—N6—N5	109.8 (3)	O1—C16—C17	120.1 (3)
C14—N6—B1	131.9 (3)	C21—C16—C17	120.2 (3)
N5—N6—B1	117.7 (2)	C18—C17—C16	118.6 (4)
C16—O1—Ir1	110.2 (2)	C18—C17—H17	120.7
C2—C1—H1A	109.5	C16—C17—H17	120.7
C2—C1—H1B	109.5	C17—C18—C19	122.7 (4)
H1A—C1—H1B	109.5	C17—C18—H18	118.7
C2—C1—H1C	109.5	C19—C18—H18	118.7
H1A—C1—H1C	109.5	C20—C19—C18	119.3 (4)
H1B—C1—H1C	109.5	C20—C19—H19	120.4
N1—C2—C3	108.7 (3)	C18—C19—H19	120.4
N1—C2—C1	124.7 (3)	C19—C20—C21	120.0 (4)
C3—C2—C1	126.5 (3)	C19—C20—H20	120.0
C4—C3—C2	106.9 (3)	C21—C20—H20	120.0
C4—C3—H3	126.6	C16—C21—C20	119.3 (3)
C2—C3—H3	126.6	C16—C21—C22	113.1 (3)
N2—C4—C3	107.9 (3)	C20—C21—C22	127.3 (3)
N2—C4—C5	123.4 (3)	C21—C22—C23	119.1 (3)
C3—C4—C5	128.7 (3)	C21—C22—Ir1	112.6 (2)
C4—C5—H5A	109.5	C23—C22—Ir1	127.8 (3)
C4—C5—H5B	109.5	C22—C23—H23A	109.5
H5A—C5—H5B	109.5	C22—C23—H23B	109.5
C4—C5—H5C	109.5	H23A—C23—H23B	109.5
H5A—C5—H5C	109.5	C22—C23—H23C	109.5
H5B—C5—H5C	109.5	H23A—C23—H23C	109.5
C7—C6—H6A	109.5	H23B—C23—H23C	109.5
C7—C6—H6B	109.5	Cl3—C24—Cl2	111.8 (3)
H6A—C6—H6B	109.5	Cl3—C24—Cl4	108.0 (3)
C7—C6—H6C	109.5	Cl2—C24—Cl4	111.2 (3)
H6A—C6—H6C	109.5	Cl3—C24—H24	108.6
H6B—C6—H6C	109.5	Cl2—C24—H24	108.6
N3—C7—C8	108.6 (3)	Cl4—C24—H24	108.6
			
C22—Ir1—N1—C2	45.2 (3)	C1—C2—C3—C4	179.5 (3)
N3—Ir1—N1—C2	143.5 (3)	N1—N2—C4—C3	−0.6 (4)
O1—Ir1—N1—C2	−37.2 (3)	B1—N2—C4—C3	−176.4 (3)
N5—Ir1—N1—C2	−129.2 (3)	N1—N2—C4—C5	179.9 (3)
C22—Ir1—N1—N2	−142.2 (2)	B1—N2—C4—C5	4.2 (5)
N3—Ir1—N1—N2	−43.9 (2)	C2—C3—C4—N2	0.3 (4)
O1—Ir1—N1—N2	135.5 (2)	C2—C3—C4—C5	179.7 (3)
N5—Ir1—N1—N2	43.4 (2)	N4—N3—C7—C8	−0.4 (4)
C2—N1—N2—C4	0.6 (3)	Ir1—N3—C7—C8	−178.1 (2)
Ir1—N1—N2—C4	−173.9 (2)	N4—N3—C7—C6	178.2 (3)
C2—N1—N2—B1	176.9 (3)	Ir1—N3—C7—C6	0.5 (5)
Ir1—N1—N2—B1	2.4 (4)	N3—C7—C8—C9	−0.1 (4)
N4—B1—N2—C4	−129.0 (3)	C6—C7—C8—C9	−178.7 (3)
N6—B1—N2—C4	111.9 (4)	N3—N4—C9—C8	−0.8 (4)
N4—B1—N2—N1	55.6 (4)	B1—N4—C9—C8	173.4 (3)
N6—B1—N2—N1	−63.4 (4)	N3—N4—C9—C10	−178.9 (3)
C22—Ir1—N3—C7	−48.8 (3)	B1—N4—C9—C10	−4.6 (6)
N1—Ir1—N3—C7	−141.8 (3)	C7—C8—C9—N4	0.6 (4)
N5—Ir1—N3—C7	132.5 (3)	C7—C8—C9—C10	178.4 (4)
Cl1—Ir1—N3—C7	44.5 (3)	N6—N5—C12—C13	0.1 (3)
C22—Ir1—N3—N4	133.7 (2)	Ir1—N5—C12—C13	−175.2 (2)
N1—Ir1—N3—N4	40.7 (2)	N6—N5—C12—C11	179.6 (3)
N5—Ir1—N3—N4	−45.0 (2)	Ir1—N5—C12—C11	4.4 (5)
Cl1—Ir1—N3—N4	−133.0 (2)	N5—C12—C13—C14	−0.1 (4)
C7—N3—N4—C9	0.8 (4)	C11—C12—C13—C14	−179.6 (3)
Ir1—N3—N4—C9	178.9 (2)	N5—N6—C14—C13	−0.1 (4)
C7—N3—N4—B1	−174.2 (3)	B1—N6—C14—C13	170.8 (3)
Ir1—N3—N4—B1	4.0 (4)	N5—N6—C14—C15	178.1 (3)
N6—B1—N4—C9	−115.4 (4)	B1—N6—C14—C15	−11.1 (5)
N2—B1—N4—C9	126.7 (3)	C12—C13—C14—N6	0.1 (4)
N6—B1—N4—N3	58.3 (4)	C12—C13—C14—C15	−177.9 (3)
N2—B1—N4—N3	−59.6 (4)	Ir1—O1—C16—C21	−10.4 (4)
N3—Ir1—N5—C12	−143.5 (3)	Ir1—O1—C16—C17	174.7 (3)
N1—Ir1—N5—C12	127.0 (3)	O1—C16—C17—C18	175.3 (4)
O1—Ir1—N5—C12	34.1 (3)	C21—C16—C17—C18	0.4 (6)
Cl1—Ir1—N5—C12	−52.4 (3)	C16—C17—C18—C19	−1.4 (7)
N3—Ir1—N5—N6	41.6 (2)	C17—C18—C19—C20	1.1 (7)
N1—Ir1—N5—N6	−47.8 (2)	C18—C19—C20—C21	0.3 (7)
O1—Ir1—N5—N6	−140.8 (2)	O1—C16—C21—C20	−174.0 (3)
Cl1—Ir1—N5—N6	132.8 (2)	C17—C16—C21—C20	0.9 (5)
C12—N5—N6—C14	0.0 (3)	O1—C16—C21—C22	0.0 (5)
Ir1—N5—N6—C14	176.2 (2)	C17—C16—C21—C22	174.8 (3)
C12—N5—N6—B1	−172.3 (3)	C19—C20—C21—C16	−1.2 (6)
Ir1—N5—N6—B1	3.9 (3)	C19—C20—C21—C22	−174.2 (4)
N4—B1—N6—C14	128.6 (3)	C16—C21—C22—C23	−161.3 (3)
N2—B1—N6—C14	−112.9 (4)	C20—C21—C22—C23	12.0 (5)
N4—B1—N6—N5	−61.1 (4)	C16—C21—C22—Ir1	11.4 (4)
N2—B1—N6—N5	57.4 (4)	C20—C21—C22—Ir1	−175.3 (3)
C22—Ir1—O1—C16	13.0 (2)	N3—Ir1—C22—C21	164.6 (2)
N1—Ir1—O1—C16	105.7 (2)	N1—Ir1—C22—C21	−105.8 (2)
N5—Ir1—O1—C16	−168.5 (2)	O1—Ir1—C22—C21	−13.0 (2)
Cl1—Ir1—O1—C16	−80.6 (2)	Cl1—Ir1—C22—C21	73.1 (2)
N2—N1—C2—C3	−0.4 (4)	N3—Ir1—C22—C23	−23.6 (3)
Ir1—N1—C2—C3	172.8 (2)	N1—Ir1—C22—C23	66.1 (3)
N2—N1—C2—C1	−179.9 (3)	O1—Ir1—C22—C23	158.9 (3)
Ir1—N1—C2—C1	−6.7 (5)	Cl1—Ir1—C22—C23	−115.1 (3)
N1—C2—C3—C4	0.1 (4)		

### Hydrogen-bond geometry (Å, º)

**Table:**

*D*—H···*A*	*D*—H	H···*A*	*D*···*A*	*D*—H···*A*
C24—H24···Cl1	1.00	2.55	3.488 (5)	156
C11—H11*A*···O1	0.98	2.37	3.230 (4)	146
C11—H11*C*···Cl3^i^	0.98	2.65	3.609 (4)	166

Symmetry code: (i) *x*, −*y*+1/2, *z*−1/2.
